# Fish predation on corals promotes the dispersal of coral symbionts

**DOI:** 10.1186/s42523-021-00086-4

**Published:** 2021-03-22

**Authors:** Carsten G. B. Grupstra, Kristen M. Rabbitt, Lauren I. Howe-Kerr, Adrienne M. S. Correa

**Affiliations:** BioSciences at Rice, Rice University, 6100 Main St, MS-140, Houston, TX 77005 USA

**Keywords:** Butterflyfish, Corallivore, Coral reefs, Dispersal, Feces, Filefish, Microbiome, Predation, Parrotfish, Sediment, Surgeonfish, Symbiodiniaceae, Water column

## Abstract

**Background:**

The microbiomes of foundation (habitat-forming) species such as corals and sponges underpin the biodiversity, productivity, and stability of ecosystems. Consumers shape communities of foundation species through trophic interactions, but the role of consumers in dispersing the microbiomes of such species is rarely examined. For example, stony corals rely on a nutritional symbiosis with single-celled endosymbiotic dinoflagellates (family Symbiodiniaceae) to construct reefs. Most corals acquire Symbiodiniaceae from the environment, but the processes that make Symbiodiniaceae available for uptake are not resolved. Here, we provide the first comprehensive, reef-scale demonstration that predation by diverse coral-eating (corallivorous) fish species promotes the dispersal of Symbiodiniaceae, based on symbiont cell densities and community compositions from the feces of four obligate corallivores, three facultative corallivores, two grazer/detritivores as well as samples of reef sediment and water.

**Results:**

Obligate corallivore feces are environmental hotspots of Symbiodiniaceae cells: live symbiont cell concentrations in such feces are 5–7 orders of magnitude higher than sediment and water environmental reservoirs. Symbiodiniaceae community compositions in the feces of obligate corallivores are similar to those in two locally abundant coral genera (*Pocillopora* and *Porites*), but differ from Symbiodiniaceae communities in the feces of facultative corallivores and grazer/detritivores as well as sediment and water. Combining our data on live Symbiodiniaceae cell densities in feces with in situ observations of fish, we estimate that some obligate corallivorous fish species release over 100 million Symbiodiniaceae cells per 100 m^2^ of reef per day. Released corallivore feces came in direct contact with coral colonies in the fore reef zone following 91% of observed egestion events, providing a potential mechanism for the transfer of live Symbiodiniaceae cells among coral colonies.

**Conclusions:**

Taken together, our findings show that fish predation on corals may support the maintenance of coral cover on reefs in an unexpected way: through the dispersal of beneficial coral symbionts in corallivore feces. Few studies examine the processes that make symbionts available to foundation species, or how environmental reservoirs of such symbionts are replenished. This work sets the stage for parallel studies of consumer-mediated microbiome dispersal and assembly in other sessile, habitat-forming species.

**Supplementary Information:**

The online version contains supplementary material available at 10.1186/s42523-021-00086-4.

## Introduction

**Fig. 1:**
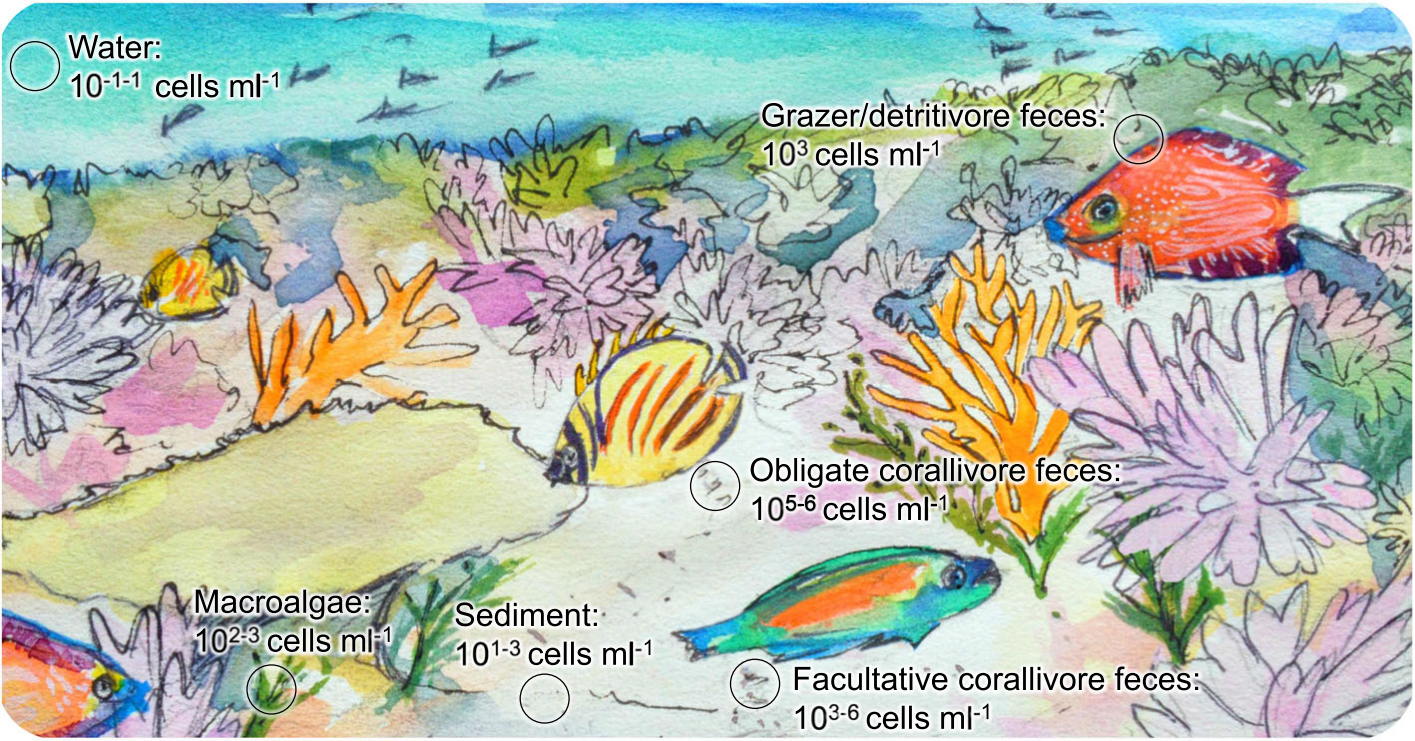
Summary of the Symbiodiniaceae cell densities quantified from coral reef environmental reservoirs in this and other studies. Cell densities reported in this study (all obligate corallivore and grazer/detritivore feces data and some data for facultative corallivore feces, sediments and seawater) represent live cell densities only, based on results from hemocytometry with the cell viability dye, trypan blue. Previously published cell densities for sediment and seawater [22, 35], macroalgae [22], and facultative corallivore feces [14] may include both dead and live cells and were quantified using hemocytometry [14], a combination of flow-cytometry and hemocytometry [35], or quantitative PCR [22]

Most animals and plants studied to date host resident communities of microorganisms— microbiomes—that influence their health and survival [7, 38]. Many hosts establish or modify their microbiome by taking up microorganisms from the environment [41]. Stony corals, for example, harbor single-celled dinoflagellates in the family Symbiodiniaceae and utilize their photosynthetic products to fuel the construction of reef frameworks [44]. Approximately 75% of spawning and 10% of brooding coral species acquire their Symbiodiniaceae from the environment with each generation [5], and adult corals may take up environmental Symbiodiniaceae cells to replenish their microbiomes following abiotic stress [8, 34, 58]. However, the dispersal mechanisms that make Symbiodiniaceae cells available to prospective host corals have not been resolved (but see [15, 45]), and Symbiodiniaceae cell densities in environmental reservoirs appear relatively low (sediments: 10^1^–10^3^ cells ml^− 1^; seawater: 10^0^–10^1^ cells ml^− 1^; macroalgae: 10^2^–10^3^ cells ml^− 1^, Fig. 1 [14, 22, 35]).

Consumers (e.g., nectarivores, herbivores, predators) are known to mediate the dispersal of microbiome constituents for various host organisms. Nectar-associated yeasts and bacteria, for example, are transmitted by nectarivores [10], and fungi associated with plant mycorrhiza are dispersed in the feces of diverse herbivorous animals [64]. Corallivores (coral-eating fish and invertebrates) that feed on corals without killing them may similarly mediate the dispersal of coral microbiomes by egesting Symbiodiniaceae—and other members of the coral microbiome—in their feces as they move across reefs [43, 59]. A total of 128 fish species spanning 11 families consume corals as part (i.e., facultative corallivores) or all (i.e., obligate corallivores) of their diets, and most of them occur in the Indo-Pacific or the Red Sea [16, 57]. To date, dispersal of Symbiodiniaceae has been quantified from one fish species: the Caribbean facultative corallivorous parrotfish *Sparisoma viride* [14]. Symbiodiniaceae cells were identified in 93% of fecal samples; cell densities in most samples were similar to those in sediments (although in some samples, cell concentrations were up to 10-fold higher [14, 35]). Molecular approaches detected three Symbiodiniaceae genera from *S. viride* feces, and two of those genera (*Symbiodinium* and *Breviolum*) commonly associate with Caribbean soft and stony corals. However, *Cladocopium* and *Durusdinium*, the two other genera commonly detected in Caribbean corals, were not observed in *S. viride* feces [14]. These results provide a first indication that facultative corallivores contribute to the dispersal of some coral-associated Symbiodiniaceae genera, at cell concentrations at least comparable to sediments. Obligate corallivores, however, ingest higher abundances of coral tissue than facultative corallivores and may therefore be more important drivers of Symbiodiniaceae dispersal, but this has not been studied. We conducted the first comprehensive, reef-scale quantification of fish-mediated coral microbiome dispersal on a Pacific reef and found, surprisingly, that the feces of *both* obligate and facultative corallivorous fishes contain high densities of live Symbiodiniaceae cells. Thus, this study adds a new dimension to our understanding of the potential outcomes of predation and reveals an important dispersal mechanism for Symbiodiniaceae on Pacific reefs.

We characterized Symbiodiniaceae densities and community compositions in the feces of four obligate corallivores (the butterflyfishes *Chaetodon lunulatus*, CHLU; *Chaetodon ornatissimus*, CHOR; *Chaetodon reticulatus*, CHRE; and the filefish *Amanses scopas*, AMSC) and three facultative corallivores (the butterflyfishes *Chaetodon citrinellus*, CHCI; and *Chaetodon pelewensis*, CHPE, and the parrotfish *Chlorurus spilurus*, CHSP) from a reefscape in Mo’orea, French Polynesia [16, 18, 57]. To test whether the feces of obligate corallivores constitute ‘hotspots’ of live Symbiodiniaceae and are proximate environmental sources of Symbiodiniaceae for prospective coral host colonies, we additionally characterized Symbiodiniaceae from reef-associated sediments and water as well as the feces of two grazer/detritivores (surgeonfishes *Ctenochaetus flavicauda*, CTFL; and *Ctenochaetus striatus*, CTST). We then compared Symbiodiniaceae communities in all of these samples to symbionts harbored by locally common reef-building corals *Acropora hyacinthus*, *Pocillopora* species complex and *Porites lobata* species complex [13, 20, 21, 23]. Next, we used bootstrap models based on in situ surveys and observations to generate the first reef-scale predictions of Symbiodiniaceae dispersal for three corallivorous fish species. Finally, we quantified how often egested feces came in direct contact with coral colonies in situ to estimate how frequently fish egestion potentially influences Symbiodiniaceae community assembly in coral hosts.

## Results and discussion

### Most corallivorous fish feces contain live Symbiodiniaceae cells

A fecal sample was isolated from the hindgut of each of 76 fishes (*n* = 6–14 individuals per species; 9 species; see Table S1 for replication) so that fecal material examined had passed through the entirety of the fish digestive tract. We then measured the volume and mass of each fecal sample and examined it for the presence of live Symbiodiniaceae cells. Sediment and water samples were included as environmental controls (*n* = 12 each). We used a Neubauer hemocytometer and a light microscope, and samples were stained with Trypan Blue to identify Symbiodiniaceae cells that were live at the time of preservation (Figure S1 [60, 68]). We detected live Symbiodiniaceae cells in 100% of obligate corallivore feces (*n* = 40, across four species) and in ~ 82% of facultative corallivore feces using microscopy (*n* = 18 of 22, across three species). Live Symbiodiniaceae cells were detected by this visual method in ~ 36% of grazer/detritivore feces (*n* = 5 of 14, across two species), ~ 42% of sediment samples (*n* = 5 of 12) and ~ 8% of seawater samples (*n* = 1 of 12).

### Live Symbiodiniaceae cell concentrations are highest in obligate corallivore feces

**Fig. 2:**
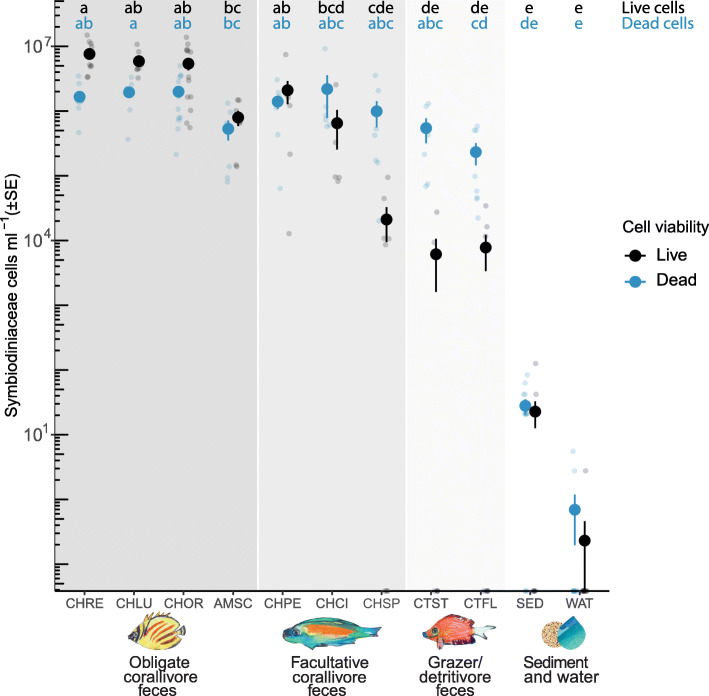
Feces of obligate corallivores (coral-eating animals) and some facultative corallivores contain live Symbiodiniaceae cells at densities several orders of magnitude higher than the feces of grazer/detritivores, sediment or water samples. Large points indicate the mean live (black) or dead (blue) Symbiodiniaceae cell density of a given species or sample type; vertical lines depict standard errors of the mean (SE). Each small point indicates cell densities quantified from a fecal sample collected from the hind gut of an individual fish (*n* = 76 total) or from an individual sediment or water sample (*n* = 24 total). Included samples (and sample sizes) for each sample category are as follows: Obligate corallivores (dark shading): *Amanses scopas*, AMSC (7); *Chaetodon lunulatus*, CHLU (8); *Chaetodon ornatissimus*, CHOR (14); *Chaetodon reticulatus*, CHRE (11). Facultative corallivores (intermediate shading): *Chaetodon pelewensis*, CHPE (8); *Chaetodon citrinellus*, CHCI (6); and *Chlorurus spilurus* CHSP (8). Grazer/Detritivores (light shading): *Ctenochaetus flavicauda*, CTFL (8); and *Ctenochaetus striatus*, CTST (6). Sediment and water (no shading): Sediment, SED (12); Water, WAT (12). Significant differences are listed in Table S2. For data used, see Additional file 2

Live Symbiodiniaceae cell densities ml^− 1^ differed among species and environmental samples (Fig. 2; Overall Kruskal-Wallis test results: chi-squared = 85.21, df = 10, *p*-value< 0.001), with higher mean live cell densities observed in the feces of each obligate corallivore species than in feces of grazer/detritivore species and sediment and water samples (Fig. 2; Table S2). Dead Symbiodiniaceae cell densities also differed among species and environmental samples, with higher concentrations of dead Symbiodiniaceae cells in corallivore feces than in environmental samples (Fig. 2; Table S2; Overall Kruskal-Wallis test results: chi-squared = 72.36, df = 10, *p*-value< 0.001).

Mean live Symbiodiniaceae cell concentrations in individual obligate corallivore species (Fig. 2) ranged from 7.96·10^5^ ± 5.15·10^5^ (*Amanses scopas*) to 7.62·10^6^ ± 2.55·10^6^ (*Chaetodon reticulatus*) ml^− 1^ of feces and were 4–5 orders of magnitude higher than sediment samples (22.75 ± 22.37) and 6–7 orders of magnitude higher than seawater samples (0.23 ± 0.51). Mean live Symbiodiniaceae cell densities in feces of facultative corallivore species were more variable than obligate corallivores and ranged from 2.11·10^4^ ± 2.76·10^4^ (*Chlorurus spilurus*) to 2.09·10^6^ ± 1.94·10^6^ (*Chaetodon pelewensis*) ml^− 1^ feces. Overall, live Symbiodiniaceae cell densities in corallivore feces in this study were 1–3 orders of magnitude higher than values previously reported from the corallivorous Carribean parrotfish *Sparisoma viride* (live and dead cells combined: 3.21·10^3^–8.90·10^3^ cells ml^− 1^ [14]). The higher cell densities observed in our study are likely due to dietary differences among fishes. In general, the corallivores in our study rely on corals for a larger part of their diets than *S. viride* [14, 24]*,* and are therefore expected to ingest higher abundances of Symbiodiniaceae cells. The exception to this is *C. spilurus* which, like *Sparisoma viride*, is a facultative corallivore. In our study, *C. spilurus* bit coral at a lower rate (~ 6%, data not shown) than was reported for *S. viride* (~ 13% bite rate, [14]). Despite this, *C. spilurus* feces still contained Symbiodiniaceae densities an order of magnitude higher than *S. viride* feces [14]. Methodological differences may explain this apparent discrepancy: Castro-Sanguino and Sánchez [14] collected feces from the benthos after egestion by *S. viride* and sieved the fecal pellets, which likely resulted in the loss of more Symbiodiniaceae cells, compared to direct sampling from fish hindguts (this study).

### Symbiodiniaceae cells in corallivore feces are competent for division and long-term survival

The long-term viability of Symbiodiniaceae cells in feces was further confirmed through culturing (attempted from 61 fish fecal pellets using 24 replicate wells per sample; 1464 wells total). Six to 10 weeks after cultures were instigated, live Symbiodiniaceae cells in coccoid, mitotic and motile life stages were documented in at least one replicate culture well from 54% of obligate corallivore feces (*n* = 19 of 35 samples), 11% of facultative corallivore feces (*n* = 2 of 18 samples), and 38% of grazer/detritivore feces (*n* = 3 of 8 samples) indicating that the feces of various reef fish consumers contain Symbiodiniaceae cells that are competent for division and long-term survival. Additional research is needed to explore the variation in symbiont culturing success detected across consumer categories, specifically whether corallivore traits and/or variation in Symbiodiniaceae diversity drove the observed differences.

### The communities of Symbiodiniaceae in obligate corallivore feces are most similar to those in locally abundant corals

**Fig. 3:**
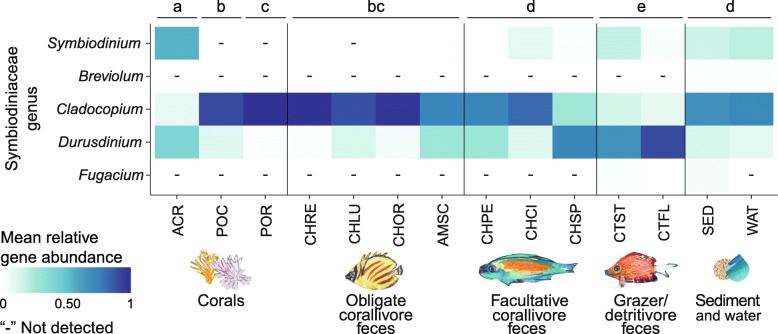
The communities of Symbiodiniaceae in obligate corallivore (coral-eating animal) feces are most similar to the communities of Symbiodiniaceae in two locally abundant coral species. Mean relative abundance of hits to a given Symbiodiniaceae genus in corals, obligate corallivore feces, facultative corallivore feces, grazer/detritivore feces, and reef-associated sediment and water (all included samples had > 1000 reads). Mean relative abundances were calculated across all samples from a species or sample category. Dashes (“-”) indicate that a Symbiodiniaceae genus was not detected in any of the samples from a fish species or sample category. Squares appearing white but without a “- “had low (≤1%) but still detectable mean relative abundances. Different letters indicate significant differences in Symbiodiniaceae community compositions based on pairwise PERMANOVA tests (*p* < 0.05, using the Benjamini-Hochberg correction for multiple comparisons) using Bray-Curtis distances based on randomly subsampled (*n* = 12) untransformed data (Table S1, S4). Overall PERMANOVA test results: df = 6, F = 17.30, *R*^2^ = 0.58, *p* = 0.001. Species and sample categories (and the fraction of samples that had > 1000 reads and were thus included in the analysis) are as follows: Corals: *Acropora hyacinthus*, Acropora (11/12); *Pocillopora* species complex, Pocillopora (12/12); *Porites lobata* species complex, Porites (12/12). Obligate corallivores: *Chaetodon reticulatus,* CHRE (11/11); *Chaetodon lunulatus,* CHLU (8/8); *Chaetodon ornatissimus,* CHOR (14/14); *Amanses scopas,* AMSC (7/7). Facultative corallivores: *Chaetodon pelewensis,* CHPE (8/8); *Chaetodon citrinellus,* CHCI (6/6); and *Chlorurus spilurus* CHSP (8/8). Grazer/Detritivores: *Ctenochaetus striatus,* CTST (6/6); and *Ctenochaetus flavicauda,* CTFL (7/8). Sediment and water: Sediment, SED (12/12); Water, WAT (7/12). For data used, see Additional file 2

To examine whether Symbiodiniaceae communities in corallivore feces constitute a potential source of symbiont cells for uptake by local corals, we characterized community compositions of Symbiodiniaceae in all our samples (fish feces, sediment and water) as well as in three common coral species (*Acropora hyacinthus*, *Pocillopora* species complex, and *Porites lobata* species complex; *n* = 12 per coral species; Table S1) by sequencing the internal transcribed spacer-2 (ITS-2) region of Symbiodiniaceae rDNA. The coral species examined here harbor unique Symbiodiniaceae communities [52, 58] and are members of coral genera frequently targeted by the corallivores in this study [50]. At the genus level, symbiont communities differed among coral species, obligate corallivore feces, grazer/detritivore feces, and facultative corallivore feces, reef sediments and seawater (PERMANOVA: df = 6, F = 17.30, R^2^ = 0.58, *p* = 0.001; Fig. 3, Figure S2). On average, sequenced reads from obligate corallivore feces were dominated by similarities to *Cladocopium* (72–98% of reads), with low relative abundances of reads identified as the genera *Durusdinium* (2–27% of reads) and *Symbiodinium* (0–1% of reads). Thus, Symbiodiniaceae communities in obligate corallivore feces overall most closely resembled symbiont compositions in the *Pocillopora* species complex and *Porites lobata* species complex corals, which mainly harbored symbionts in the genus *Cladocopium* (Table S3 and S4). This aligns with our observations that the corallivore species in this study rely on pocilloporid and poritid corals for a large part of their diets (observations in this study [50]). It also agrees with the fact that pocilloporid and poritid corals were more common at our study site at the time of sampling, compared to acroporid corals (which were dominated by *Durusdinium* and *Symbiodinium*, Fig. 3). Facultative corallivore feces were similar to sediment and seawater samples (Table S3 and S4); these feces contained, on average, less *Cladocopium* (26–81%), but more *Durusdinium* (11–73%) and *Symbiodinium* (0–8%) than obligate corallivores (Fig. 3). Grazer/detritivore feces were distinct from all other sample categories based on pairwise PERMANOVA test results (Table S4). The Symbiodiniaceae genera *Breviolum* and *Fugacium*, which are rare in Mo’orean corals [52], were detected only in grazer/detritivore feces (*Fugacium* only), reef-associated seawater (*Breviolum* only) and sediment samples (Fig. 3, S2; Table S3).

### Obligate corallivores disperse millions of live Symbiodiniaceae cells per 100m^2^ of reef per day

**Fig. 4:**
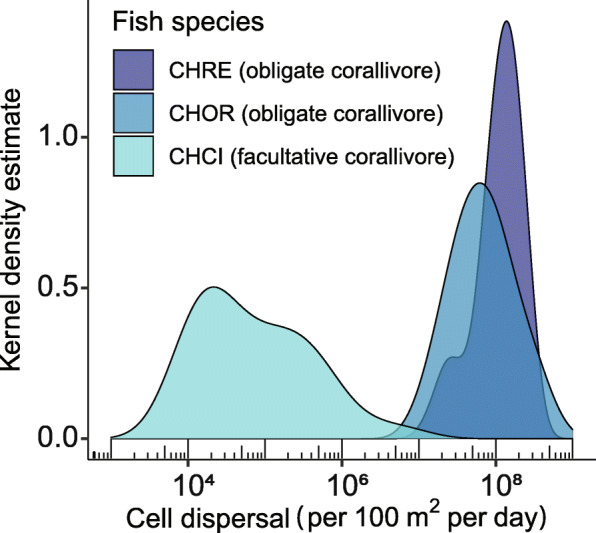
Corallivores on a South Pacific reef disperse millions of live Symbiodiniaceae cells per 100 m^2^ per day. We estimated the number of live Symbiodiniaceae cells dispersed by the obligate corallivores *Chaetodon ornatissimus* (CHOR) and *Chaetodon reticulatus* (CHRE) and the facultative corallivore *Chaetodon citrinellus* (CHCI) per 100 m^2^ per day by applying a bootstrap approach (1000 iterations) to the equation *T* = *gSWCF*. The estimated number of dispersed Symbiodiniaceae cells (*T*) is the product of five variables: A fish species-specific constant representing the estimated number of egestions in an eight-hour day (*g*); observed fecal pellet sizes in cm (*S*); measured densities of fecal samples in g cm^− 1^ (*W*); measured densities of Symbiodiniaceae cells g^− 1^ feces (*C*); and observed fish densities per 100 m^2^ (*F*). Due to variation in fish species distributions, most data for CHOR and CHRE were collected on the fore reef, whereas data for CHCI were collected on the back reef. See Table S5 for replication. For data used see Additional file 2

To generate the first estimates of the daily dispersal of live Symbiodiniaceae cells by corallivorous fish at the reef scale, we applied a bootstrap approach to the product of observed egestion rates, fecal pellet sizes (from in situ field observations), fish densities [9], and live Symbiodiniaceae cell densities and fecal pellet densities (from ex situ measurements) for two obligate corallivores (*C. ornatissimus* and *C. reticulatus*) and one facultative corallivore (*C. citrinellus*). This resulted in a probability distribution describing the likely number of live cells dispersed by each species per 100 m^2^ per day (Fig. 4). The mean (± 95% CI) estimated dispersal rates for the obligate corallivores were 1.01·10^8^ ± 6.50·10^6^ and 1.27·10^8^ ± 4.61·10^6^ cells per 100 m^2^ d^− 1^, respectively; these were three orders of magnitude higher than the estimated mean for the facultative corallivore (3.32·10^5^ ± 6.21·10^4^). Differences between obligate versus facultative corallivore estimates were mainly driven by higher densities of *C. ornatissimus* and *C. reticulatus* individuals at our study site (4.7–8.8 times higher than *C. citrinellus*, Table S5), and higher Symbiodiniaceae densities in their feces (3.7–7.5 times higher than *C. citrinellus*, Table S5, Fig. 2). Further, mean fecal pellet size, fecal density and egestion rates were also 1.1–4.5 times higher for the two obligate corallivores, relative to the facultative corallivore (Table S5).

### Corallivores promote the dispersal of live Symbiodiniaceae cells among coral colonies

Our findings indicate that obligate corallivore feces contain live Symbiodiniaceae cells at densities two to seven orders of magnitude higher than other environmental reservoirs, such as the water column, sediments and macroalgae (this work [22, 35]), and up to three orders of magnitude higher than feces of the one other corallivorous fish that has been examined: the Caribbean parrotfish *S. viride* [14]. Corallivore feces that come in direct contact with live coral colonies are likely to facilitate the transmission of Symbiodiniaceae cells between colonies. We therefore conducted surveys to estimate the relative frequency at which corallivore feces fell onto such colonies. We observed that egested feces often fell apart into several segments as they fell through the water column, and segments of ~ 91% of egested feces landed on live corals at our fore reef site (*n* = 20 of 22 feces). Segments of only 10% of feces fell onto live corals at our back reef site (*n* = 3 of 30 feces), which is consistent with lower mean (± SD) live coral cover in this reef zone (44.80% ± 10.83 coral cover on the fore reef; 15.33% ± 7.10 back reef, ANOVA, df = 1, F = 44.91, *p* < 0.001). These results indicate that fish predators of corals commonly mediate the dispersal of Symbiodiniaceae cells to prospective coral host colonies on Pacific reefs, especially in areas with relatively high coral cover. Additional work is needed to: (1) understand differences in fecal pellet integrity among corallivore species and individuals following egestion; and (2) resolve how subsequent biotic interactions and hydrodynamic conditions influence the duration of fecal contact with potential hosts. This information will support a more nuanced understanding of fecal fates following egestion by corallivorous fish and the associated potential for symbiont cell dispersal and uptake.

### Corallivore feces constitute environmental hotspots of live Symbiodiniaceae

Our findings suggest that the feces of obligate corallivores and at least some facultative corallivores constitute significant but underexplored environmental ‘hotspots’ of Symbiodiniaceae on coral reefs; such feces may supply Symbiodiniaceae cells to potential hosts directly, or to other environmental reservoirs as they disintegrate [14, 45]. Corals have been shown to take up Symbiodiniaceae from sediments and seawater, as well as from the feces of giant clams [15, 34, 45, 63]. The sea anemone *Aiptasia pulchella* has also previously been shown to incorporate Symbiodiniaceae from fish feces as symbionts [43]. Thus, although further experimental verification is needed, corals (in at least some life stages and health states) are likely capable of incorporating Symbiodiniaceae from fish feces.

We posit that corallivore feces may be particularly beneficial for juvenile corals (individuals up to 4 years post-settlement), and especially horizontal transmitters, since each generation must assemble its Symbiodiniaceae community from environment pools. Juveniles of diverse coral species are known to readily take up diverse Symbiodiniaceae species from the water column and sediments [1–3, 39, 45, 49]. Further, experiments have demonstrated that inoculations of coral juveniles with some Symbiodiniaceae species may increase post-settlement survival and growth [40, 61]. Experiments examining how contact with the feces of different fish consumer groups influences the health and survival of coral juveniles, the time to establishment and composition of their Symbiodiniaceae communities, as well as other factors (e.g., colony growth rate) constitute important next steps in exploring the ecological implications of consumer-mediated symbiont dispersal on reefs and analogous systems. The impact of feces from different fish consumer groups on coral colony health and Symbiodiniaceae community assembly in adult corals should also be tested experimentally. Limited evidence suggests that adult coral colonies may take up novel Symbiodiniaceae species during or following environmental stress [8, 34, 58], but the influence of predator feces has not been examined. A tractable first step is to test whether adult coral colonies that have lost most or all of their symbionts (i.e. bleached, [66]) recover their Symbiodiniaceae communities more quickly—or exhibit higher survival—when exposed to corallivore feces. Finally, these lines of inquiry can be extended to other members of the coral microbiome, such as bacteria, which also contribute to the health and disease of their hosts [54, 56].

The dispersal of beneficial microorganisms may become increasingly important [19, 53] as anthropogenic stressors disrupt animal and plant microbiomes, leading to disease and mortality [28, 31, 48, 67]. Our results suggest that shifts and declines in fish communities due to overfishing [6, 26, 37] and habitat degradation [51, 65] may contribute to an unexplored issue on reefs: altered (or reduced) dispersal of Symbiodiniaceae, a key member of the coral microbiome. Coral-Symbiodiniaceae partnerships have been increasingly disrupted over the past four decades, resulting in coral reef decline [28, 67]. To help corals tolerate stress and mitigate reef degradation [47], probiotic solutions of beneficial Symbiodiniaceae [42] and bacteria [55] are currently being developed. We show that corallivores egest feces containing high densities of live Symbiodiniaceae cells directly onto corals. This behavior may constitute a routine, global-scale ‘restoration effort’ that inoculates corals with natural probiotics derived from nearby colonies. It is thus important that healthy fish assemblages are maintained on reefs as the potentially stabilizing effect of corallivores on coral microbiomes is investigated.

## Conclusion

Climate change stressors, along with overfishing and nutrient pollution, are driving the loss of coral reefs globally. The roles of herbivores in maintaining a coral-dominated reef state, particularly following disturbance, are well established (e.g. [11, 12]). Our findings suggest that corallivorous fish may also contribute to the success and resilience of corals, because their feces constitute environmental hotspots of Symbiodiniaceae from which corals may acquire (or replenish) their microbiomes. The approaches used here can be broadly applied to studying the dispersal of symbiotic microorganisms via trophic interactions and set the stage for parallel studies of consumer-mediated microbiome dispersal and assembly in other sessile, habitat forming species.

## Methods

Individuals of nine fish species, as well as corals, sediments, and seawater samples (*n* = 6–14 per sample type or species, see Table S1) were collected on scuba in July and August 2019 from two reef zones: the back reef (1–2 m depth) and fore reef (5–10 m depth), between LTER sites 1 and 2 of the Mo’orea Coral Reef (MCR) Long Term Ecological Research (LTER) site. We selected fish species that broadly differ in their level of corallivory based on published literature [18, 24, 57, 65] as obligate corallivores (butterflyfishes *Chaetodon lunulatus*, *Chaetodon ornatissimus, Chaetodon reticulatus,* and the filefish *Amanses scopas*), facultative corallivores (butterflyfishes *Chaetodon citrinellus* and *Chaetodon pelewensis* and the parrotfish *Chlorurus spilurus*) and grazer/detritivores (surgeonfishes *Ctenochaetus flavicauda* and *Ctenochaetus striatus*). We sampled three coral species (*Acropora hyacinthus*, *Pocillopora* species complex [23], and *Porites lobata* species complex [20, 21]) that are dominant reef builders in Mo’orea and that are members of genera frequently targeted by corallivores [13, 50]. These coral species harbor unique Symbiodiniaceae communities [52, 58].

### Sample processing

Following collection, all fish were euthanized and immediately transported on ice to the lab for Symbiodiniaceae density, viability, and community composition analyses. For each collected fish, an incision was made from the pelvic fin to the anus using a sterile scalpel, and the entire digestive tract was removed. Feces were subsequently sampled from the hindgut of each fish individual (*n* = 6–14 per species) so that Symbiodiniaceae condition (live versus dead) was assessed from cells that had passed through the entire fish digestive tract. Characteristics of fecal material were also measured to support reef-scale estimates of Symbiodiniaceae cell dispersal. Fecal samples for Symbiodiniaceae cell counts were preserved in 750 μl 10% formalin in 100 kDa-filtered seawater and weighed using a microbalance immediately after dissection out of each fish (sample weights: range = 22 to 170 mg; μ = 73 mg, σ = 32). Samples for DNA extractions were preserved in DNA/RNA shield (Zymo Research, CA). To calculate fecal densities for reef scale dispersal estimates, another fecal sub-sample was weighed and its length measured.

Six colonies of each coral species were sampled from the tips of their branches (*Acropora hyacinthus* and *Pocillopora* species complex) or from the center of the colony (*Porites lobata* species complex) at both the fore reef and back reef site using bone cutters. All coral samples were taken from colonies that were at least 5 m apart. Each coral fragment was preserved for DNA extractions in DNA/RNA shield (Zymo Research, CA); samples for Symbiodiniaceae cell density analyses were not collected from corals.

Sediment and seawater samples were collected and processed using a protocol modified from Littman et al. [35]. Seawater samples (3.8 l each) were collected from the fore reef and back reef (*n* = 6 per reef zone) at a distance of 10–100 cm off the reef bottom (at least 10 m apart). The samples were concentrated to ~ 100 ml using tangential flow filtration (0.2 μm), divided into two 50 ml subsamples and filtered onto two separate 0.45-μm filters using a vacuum pump. One filter per sample was preserved for Symbiodiniaceae cell counts by resuspension in 3 ml 5% formalin in 100 kDa-filtered seawater and vortexing at maximum speed for 10 min. The other filter was preserved for the isolation of genomic material in 6 ml of DNA/RNA shield (Zymo Research, CA) with 1.35 g Lysing Matrix A garnet and 5 0.6-cm ceramic beads (MPBio, California) and vortexed for 20 min.

Pooled reef sediment samples (totaling 500 ml each) were collected concomitantly with water column samples (*n* = 6 per reef zone). Samples were pooled from ten sterile 50 ml conical tubes filled at five random locations in a 10 m radius from where the associated seawater sample was collected. To best approximate the Symbiodiniaceae cells that would be available for uptake by nearby coral colonies, sediment collections were focused at the sediment-water interface (no deeper than 5 cm) and occurred less than 1 m from live coral colonies. Sediments were immediately washed over a 120-μm nylon mesh using 500 ml 100 KDa filtered seawater and filtered through a 20-μm nylon mesh. The flow-through was then processed in the same manner as described above for seawater samples.

### Symbiodiniaceae cell density and viability

All cell count samples were homogenized using a Fisher Scientific homogenizer F150 for 5 s (to break up cell clumps) and subsequently filtered through 120 and 20 μm nylon filters to reduce the density of debris in samples. The volume of each fecal sample was measured using a 1 ml pipettor (range = 42 to 234 μl, μ = 109 μl, σ = 45). Symbiodiniaceae cells in fish feces, seawater and sediments were manually quantified (the mean of eight technical replicates) using light microscopy (20X) with a Neubauer hemocytometer. During counts, cells were counted as Symbiodiniaceae if they were of the correct size (6–12 μm [32]), had visible organelles, a coccoid shape, and a large pyrenoid accumulation body [35, 45]. Subsamples were frequently viewed at higher magnification to verify their identity, using cultured Symbiodiniaceae cells (live and dead) as visual references. Cell densities were expressed as cells per ml source material (feces, water, sediment) by dividing the cell concentration by the fraction of sample in the summed volume of fixative, stain and sample volume, and multiplying this by 1000. All samples were stained with 0.16% trypan blue 5 min before processing to parse live from dead cells [25, 60, 68]. Individual cells were interpreted as having been ‘live’ at the time of fixation if, following staining, they retained a golden-brown color (see figure S1 in the Supplementary Materials). Cells were interpreted as ‘dead’ at the time of fixation if staining turned them blue. We did not detect live Symbiodiniaceae cells in some *C. spilurus* feces, some grazer/detritivore feces and in some sediment and water samples. To avoid overinflating live Symbiodiniaceae cell density averages for these sample types, we entered cell concentrations of 0 cells ml^− 1^ for these samples, rather than excluding samples below the detection limit.

We utilized cell culturing to confirm that Symbiodiniaceae cells in fish feces could remain viable for an extended period following passage through a consumer digestive tract culturing (fecal pellets from *n* = 61 individual fish, 24 replicate wells per sample; 1464 wells total; Table S1). Briefly, subsamples of fecal pellets were suspended in 1 ml of 100KDa-filtered seawater in a sterile petri dish. Then, 20 ul of this suspension was pipetted into each of 24 replicate wells in a 96-well plate filled with 200 ul sterile F/2 media. The culture plates were stored at room temperature under fluorescent lighting on a ~ 12:12 light:dark cycle for up to 6 weeks and then transported back to Rice University (Houston, TX, USA), where they were transferred to an incubator set to 25 °C on a 12:12 light:dark cycle. All culture wells were examined after 6–10 weeks using a compound microscope with 20x and 40x magnification and scored for the presence/absence of intact Symbiodiniaceae cells.

### Symbiodiniaceae community composition

DNA was extracted using the ZymoBIOMICs DNA/RNA Miniprep kit (Zymo Research, CA), and the internal transcribed spacer-2 (ITS-2) region of Symbiodiniaceae rDNA was sequenced following Howe-Kerr et al. [27] using the primers SYM_VAR_5.8SII (5′ GAATTGCAGAACTCCGTGAACC 3′) and SYM_VAR_REV (5′ CGGGTTCWCTTGTYTGACTTCATGC 3′) [30] and sequenced on the Illumina MiSeq platform at the Georgia Genomics and Bioinformatics Core (University of Georgia, Athens, GA) following details outlined in Howe-Kerr et al. [27]. The resulting sequencing data were processed using Symportal [29]. Samples with < 1000 reads were discarded and sequencing depth was assessed using rarefaction curves. To circumvent issues related to the interpretation of inter- versus intragenomic variation in the Symbiodiniaceae ITS-2 region [17, 33], ITS2-profiles were reduced to number of reads per Symbiodiniaceae genus and expressed as percentages.

### Reef-scale Symbiodiniaceae dispersal estimates

To generate the first estimates of daily dispersal of live Symbiodiniaceae cells by corallivorous fish at the reef scale, we estimated the number of Symbiodiniaceae cells dispersed by the obligate corallivores *C. ornatissimus* and *C. reticulatus* and the facultative corallivore *C. citrinellus* per 100 m^2^ per day by applying a bootstrap approach (1000 iterations) to the equation *T* = *gSWCF*. Here, the estimated number of dispersed Symbiodiniaceae cells (*T*) is the product of five variables: A species-specific constant representing the estimated number of egestions in an eight-hour day (*g*); fecal pellet sizes in cm (*S*); densities of fecal samples in g cm^− 1^ (*W*); densities of Symbiodiniaceae cells g^− 1^ feces (*C*); and fish densities 100 m^− 2^ (*F*).

We collected data on fish egestion rates (*g*) and fecal pellet sizes (*S*) in situ via fish follows in the field between 09:00 h and 17:00 h (Table S6). Individuals of *C. ornatissimus* (*n* = 53), *C. reticulatus* (*n* = 39) and *C. citrinellus* (*n* = 31), were each followed for a mean of 7.9 min (16.2 h of observations total) and all egestion events were recorded. Estimated egestion rates per hour were calculated as the total number of observed egestions divided by the total period of observations per species*.* We conservatively assumed that the observed fish species were active for 8 h per day, equal to the time frame over which we made our observations, and that egestion rates remained constant throughout the day. We measured the length of any fecal pellet that fell on a flat surface and remained intact (*n* = 12 total) using a standard ruler. To convert the lengths of fecal pellets to weights (*W*), we used a precision balance to weigh pre-measured fecal samples from collected individuals of *C. citrinellus* (*n* = 4), *C. ornatissimus* (*n* = 3) and *C. reticulatus* (*n* = 3). Cell densities (*C*) calculated as described above were expressed as live cells g^− 1^ feces.

Fish densities (*F*) were extracted from the MCR LTER dataset (http://mcrlter.msi.ucsb.edu/cgi-bin/showDataset.cgi?docid=knb-lter-mcr.6 accessed February 14, 2020). We used fish densities from the fore reef for *Chaetodon ornatissimus* and *Chaetodon reticulatus* and data from back reef sites for *Chaetodon citrinellus*, because the latter species does not occur on the fore reef. Data from 2019 were not available as of April 2020, so we therefore used data from 2018. Briefly, the MCR LTER collects these data by counting fishes in four replicate 5 × 50 m fish belt transects at the two north-shore LTER sites (LTER 1 and 2), resulting in densities of fish 250 m^− 2^ [9]. In this study, we multiplied the LTER data by 0.4 to convert densities to 100 m^− 2^.

### Coral cover transects

Replicate line transects were conducted at our fore reef (*n* = 5) and back reef sites (*n* = 12) to determine live coral cover. Briefly, a 50 m transect was rolled out parallel to the shoreline and digital images were taken using an Olympus Tough TG6 camera to categorize substrates as “live coral” or “other” at each meter mark. These categorizations were used to calculate average percentages of live coral cover.

### Statistical analyses

For all tests, biological replicates were defined as samples collected from distinct fish individuals; or samples from coral colonies separated by at least 5 m; or sediment or water samples collected at least 10 m apart. Technical replicates (e.g., in Symbiodiniaceae cell counts) were defined as analyses conducted on multiple sub-samples of an individual biological replicate and not included in the statistical analysis.

We tested for differences in live and dead Symbiodiniaceae cell densities among individual fish species, coral species, and sediment and seawater samples. Briefly, we used Kruskal-Wallis tests and Dunn tests with Bonferroni-corrected *p*-values for multiple comparisons (Tables S2&S3) using dunn.test package v1.3.5. in R version 3.6.1 [62] because nested linear models violated the model assumptions, even after transformation. However, nested linear models produced the same general results (not shown). We checked for outliers using boxplots and observed one outlier among the facultative corallivore fecal samples. However, this datapoint was in the same range as data for obligate corallivores and was therefore retained.

We used a PERMANOVA in Vegan 2.5–6 [46] to test for differences in genus-level Symbiodiniaceae community compositions among obligate corallivores, facultative corallivores, grazer/detritivores, individual coral species, and sediments and seawater. We found significant heterogeneous dispersion between sample categories using betadisper(). Because PERMANOVA is sensitive to heterogeneous dispersion in concert with an unbalanced design [4], we randomly subsampled 12 rows for each of the four sample categories (All coral samples; Obligate corallivores: 19AMSC4, 19AMSC6, 19AMSC7, 19CHLU2, 19CHLU6, 19CHOR5, 19CHOR7, 19CHOR8, 19CHOR12, 19CHOR14, 19CHRE9, 19CHRE11; facultative corallivores: 19CHCI2, 19CHCI3, 19CHCI4, 19CHPE1, 19CHPE3, 19CHPE4, 19CHPE5, 19CHPE6, 19CHPE8, 19CHSP2, 19CHSP4, 19CHSP7; Grazer/detritivores: 19CTFL1, 19CTFL3, 19CTFL4, 19CTFL5, 19CTFL6, 19CTFL7, 19CTFL8, 19CTST1, 19CTST3, 19CTST4, 19CTST5, 19CTST6; sediment and water: BAKSED1, BAKSED2, BAKSED4, BAKSED5, FORSED1, FORSED2, FORSED3, FORSED4, FORSED6, FORH2O2, BAKH2O5, FORH2O5). We then tested for differences between sample categories using PERMANOVA with adonis() based on untransformed Bray-Curtis distances. Pairwise tests were conducted with a Benjamini-Hochberg error correction using pairwiseAdonis 0.4 [36].

To test for differences in live coral cover between the back reef and fore reef zones, we used an analysis of variance (ANOVA). We tested for normality of the distributions and of the residuals using a Shapiro test. The assumption of homogeneity of variance was tested using Levene’s test.

## Supplementary Information

**Additional file 1: Table S1.** Summary of the samples collected and processed in this study for Symbiodiniaceae cell density and viability (Fig. 2), culturing, and community composition based on the internal transcribed spacer-2 (ITS-2) region of rDNA (Fig. 3). **Figure S1.** Hemocytometry with trypan blue stain accurately differentiates live and dead Symbiodiniaceae cells that were fixed in 10% formalin. **Table S2.** Results of pairwise Dunn tests on Symbiodiniaceae cell densities between species, sediment and water samples (Fig. 2). **Table S3.** Mean relative abundances of genes associated with five Symbiodiniaceae genera identified in this study per sample type (e.g., *Amanses scopas* feces) and overall sample category (e.g., obligate corallivores) (Fig. 3). **Table S4.** Results from pairwise PERMANOVA tests on Symbiodiniaceae community composition at the genus level, based on Bray-Curtis distances (Fig. 3). **Figure S2.** The communities of Symbiodiniaceae in obligate corallivore (coral-eating animal) feces are most similar to the communities of Symbiodiniaceae in two locally abundant coral species. **Table S5.** Overview of values used in bootstrap estimate of reef-scale Symbiodiniaceae dispersal (Fig. 4).

**Additional file 2: Source data.** The tabs “cell counts_metadata” and “Cellcounts” contain densities of live (unstained by trypan blue; column ‘Live’) and dead (stained by trypan blue; column ‘Dead’) Symbiodiniaceae cells ml^− 1^ material (feces, sediment seawater), as determined through cell counts using a hemocytometer and compound microscope at 20x magnification. Reported cell densities are averages of 8 replicate counts. The code to replicate this analysis can be found on https://github.com/CorreaLab/Fishces_2020/blob/master/Symbiodiniaceae%20cell%20counts%20code. The tabs starting with “ITS2” contain sequencing data after processing in Symportal [29]. Individual ITS-2 profiles have been removed to facilitate analysis of Symbiodiniaceae communities at the genus level. All tabs except the “ITS2 metadata” tab are required for analysis using code deposited on https://github.com/CorreaLab/Fishces_2020/blob/master/Symbiodiniaceae%20community%20analysis%20code. The tab “ITS2 random subsampling” contains the randomly subsampled data used for statistical analysis with PERMANOVA. The tabs “Bootstrap_datafile_metadata” and “Bootstrap_datafile” contain the dataset used to estimate the daily dispersal of Symbiodiniaceae cells by three fish species. The column ‘Live_g_sample’ contains Symbiodiniaceae cell densities g^− 1^ fecal sample; the column ‘Wet_weight’ contains the weight of fecal pellets cm^− 1^; the columns ‘MCR_LTER_Site’ – ‘Fish_density’ contain fish abundances at four transects of MCR LTER sites 1 and 2; the column ‘Pellet_length’ contains lengths of fecal pellets as measured during observations in the field. The code to replicate this analysis is available on https://github.com/CorreaLab/Fishces_2020/blob/master/Symbiodiniaceae%20cell%20dispersal%20bootstrap%20code

## Data Availability

Cell counts data have been uploaded to Dryad (doi:10.5061/dryad.80gb5mkpd). Raw amplicon sequencing data have been deposited on NCBI’s SRA (sequence read archive; accession PRJNA655793). Source data files have been provided for figures 2-4 in “Additional file 2”. All code used for analyses is available on Github https://github.com/CorreaLab/Fishces_2020.

## Abbreviations

ANOVA: Analysis of variance
CI: Confidence interval
ITS-2: Internal transcribed spacer-2
LTER: Long-term Ecological Research
MCR: Mo’orea Coral Reef
PERMANOVA: Permutational multivariate analysis of variance
SD: Standard deviation
SE: Standard error of the mean

## Species and sample types

ACR: *Acropora hyacinthus* (stony coral)
POC: *Pocillopora* species complex (stony coral)
POR: *Porites lobata* species complex (stony coral)
AMSC: *Amanses scopas* (obligate corallivorous filefish)
CHCI: *Chaetodon citrinellus* (facultative corallivorous butterflyfish)
CHLU: *Chaetodon lunulatus* (obligate corallivorous butterflyfish)
CHOR: *Chaetodon ornatissimus* (obligate corallivorous butterflyfish)
CHPE: *Chaetodon pelewensis* (facultative corallivorous butterflyfish)
CHRE: *Chaetodon reticulatus* (obligate corallivorous butterflyfish)
CHSP: *Chlorurus spilurus* (facultative corallivorous parrotfish)
CTFL: *Ctenochaetus flavicauda* (grazer/detritivore surgeonfish)
CTST: *Ctenochaetus striatus* (grazer/detritivore surgeonfish)
SED: Sediment
WAT: Water
