# Supplementary material for: Fish predation on corals promotes the dispersal of coral symbionts
Source: Anim Microbiome. 2021 Mar 22;3:25. doi: 10.1186/s42523-021-00086-4 (PMC7986512; doi:10.1186/s42523-021-00086-4)
Supplement: Supplementary file 1 — Additional file 1: Table S1. Summary of the samples collected and processed in this study for Symbiodiniaceae cell density and viability (Fig. 2), culturing, and community composition based on the internal transcribed spacer-2 (ITS-2) region of rDNA (Fig. 3). Figure S1. Hemocytometry with trypan blue stain accurately differentiates live and dead Symbiodiniaceae cells that were fixed in 10% formalin. Table S2. Results of pairwise Dunn tests on Symbiodiniaceae cell densities between species, sediment and water samples (Fig. 2). Table S3. Mean relative abundances of genes associated with five Symbiodiniaceae genera identified in this study per sample type (e.g., Amanses scopas feces) and overall sample category (e.g., obligate corallivores) (Fig. 3). Table S4. Results from pairwise PERMANOVA tests on Symbiodiniaceae community composition at the genus level, based on Bray-Curtis distances (Fig. 3). Figure S2. The communities of Symbiodiniaceae in obligate corallivore (coral-eating animal) feces are most similar to the communities of Symbiodiniaceae in two locally abundant coral species. Table S5. Overview of values used in bootstrap estimate of reef-scale Symbiodiniaceae dispersal (Fig. 4). [file 42523_2021_86_MOESM1_ESM.docx]

**Table S1: Summary of the samples collected and processed in this study for Symbiodiniaceae cell density and viability (Figure 2), culturing, and community composition based on the internal transcribed spacer-2 (ITS-2) region of rDNA (Figure 3).** *Pocillopora* spp. = *Pocillopora* species complex; *Porites lobata* spp. = *Porites lobata* species complex.

| Sample category and type | Code | Symbiodiniaceae cell density & viability | | | Symbiodiniaceae culturing | | | Symbiodiniaceae community composition  (>1,000 ITS-2 reads) | | |
| --- | --- | --- | --- | --- | --- | --- | --- | --- | --- | --- |
|  |  | Back reef | Fore reef | **total** | Back reef | Fore reef | **total** | Back reef | Fore reef | **total** |
| **Obligate corallivores** |  |  |  |  |  |  |  |  |  |  |
| *Amanses scopas* | AMSC | 0 | 7 | **7** | 0 | 6 | **6** | 0 | 7 | **7** |
| *Chaetodon lunulatus* | CHLU | 7 | 1 | **8** | 5 | 1 | **6** | 7 | 1 | **8** |
| *Chaetodon ornatissimus* | CHOR | 6 | 8 | **14** | 6 | 7 | **13** | 6 | 8 | **14** |
| *Chaetodon reticulatus* | CHRE | 3 | 8 | **11** | 3 | 7 | **10** | 3 | 8 | **11** |
| Category sum |  | 16 | 24 | **40** | 14 | 21 | **35** | 16 | 24 | **40** |
|  |  |  |  |  |  |  |  |  |  |  |
| **Facultative corallivores** | |  |  |  |  |  |  |  |  |  |
| *Chaetodon citrinellus* | CHCI | 6 | 0 | **6** | 6 | 0 | **6** | 6 | 0 | **6** |
| *Chaetodon pelewensis* | CHPE | 0 | 8 | **8** | 0 | 4 | **4** | 0 | 8 | **8** |
| *Chlorurus spilurus* | CHSP | 0 | 8 | **8** | 0 | 8 | **8** | 0 | 8 | **8** |
| Category sum |  | 6 | 16 | **22** | 6 | 12 | **18** | 6 | 16 | **22** |
|  |  |  |  |  |  |  |  |  |  |  |
| **Grazer/detritivores** |  |  |  |  |  |  |  |  |  |  |
| *Ctenochaetus flavicauda* | CTFL | 0 | 8 | **8** | 0 | 8 | **8** | 0 | 7 | **7** |
| *Ctenochaetus striatus* | CTST | 6 | 0 | **6** | - | - | - | 6 | 0 | **6** |
| Category sum |  | 6 | 8 | **14** | 0 | 8 | **8** | 6 | 7 | **13** |
|  |  |  |  |  |  |  |  |  |  |  |
| **Sediment and water** |  |  |  |  |  |  |  |  |  |  |
| Sediment | SED | 6 | 6 | **12** | - | - | - | 6 | 6 | **12** |
| Water | WAT | 6 | 6 | **12** | - | - | - | 3 | 4 | **7** |
| Category sum |  | 12 | 12 | **24** | - | - | **-** | 9 | 10 | **19** |
|  |  |  |  |  |  |  |  |  |  |  |
| **Corals** |  |  |  |  |  |  |  |  |  |  |
| *Acropora hyacinthus* | ACR | - | - | - | - | - | - | 6 | 5 | **11** |
| *Pocillopora* spp. | POC | - | - | - | - | - | - | 6 | 6 | **12** |
| *Porites lobata* spp. | POR | - | - | - | - | - | - | 6 | 6 | **12** |
| Category sum |  | - | - | **-** | - | - | **-** | 18 | 17 | **35** |


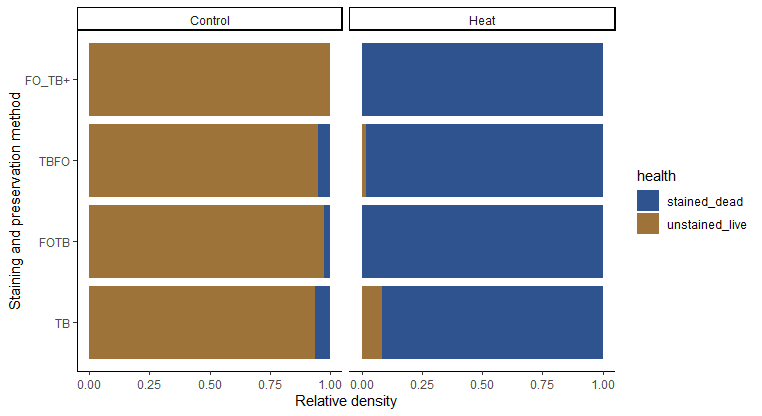


Cell viability

**Figure S1:** **Hemocytometry with trypan blue stain accurately differentiates live and dead Symbiodiniaceae cells that were fixed in 10% formalin.** We conducted a replicated experiment to confirm that hemocytometry of formalin-fixed samples in conjunction with the trypan blue stain assay accurately parses dead and live Symbiodiniaceae cells. Cultures of three Symbiodiniaceae strains (*Breviolum* sp. *Mf1.5b, Symbiodinium microadriaticum,* and *Fugacium kawagutii*) were sampled and half of each sample was kept at room temperature (Control) or exposed to 80˚C for one hour (Heat) similar to (Franklin et al., 2006). The following staining and preservation methods were tested: FO_TB+: immediately fixed in 10% formalin and stained with trypan Blue after 48 hours; TBFO: First stained with trypan blue and fixed with formalin after five minutes; FOTB: Fixed with 10% formalin and stained with trypan blue immediately afterward; TB: Stained with trypan blue and not fixed. The fraction of live Symbiodiniaceae cells differed between the heat and control treatment (two-way ANOVA: df=1, F=1263.5, p<0.001) but not between staining and preservation methods (df=3, F=0.2, p=0.889). These results indicate that preservation with 10% formalin and staining with trypan blue is an appropriate method for quantifying live and dead Symbiodiniaceae cells.

**Table S2: Results of pairwise Dunn tests on Symbiodiniaceae cell densities between species, sediment and water samples (Figure 2).** We controlled for false positives with the Benjamini-Hochberg procedure. Significant p-values (<0.05) are bolded. Overall Kruskal-Wallis test results: chi-squared=85.2132, df=10, p-value=0. Included samples (and sample sizes) for each sample type are as follows: Obligate corallivores (Obl): *Amanses scopas,* AMSC (7)*; Chaetodon lunulatus,* CHLU (8); *Chaetodon ornatissimus,* CHOR (14); *Chaetodon reticulatus,* CHRE (11). Facultative corallivores (Fac): *Chaetodon pelewensis,* CHPE (8); *Chaetodon citrinellus,* CHCI (6); and *Chlorurus spilurus* CHSP (8). Grazer/Detritivores (Gra): *Ctenochaetus flavicauda,* CTFL (8); and *Ctenochaetus striatus,* CTST (6). Sediment and water (Env): Sediment, SED (12); Water, WAT (12).

| Species comparison | Species diets | Z statistic | Adjusted p-value |
| --- | --- | --- | --- |
| AMSC - CHLU | Obl - Obl | -1.67 | 0.0771 |
| AMSC - CHOR | Obl - Obl | -1.52 | 0.0978 |
| AMSC - CHRE | Obl - Obl | -2.10 | **0.0363** |
| CHLU - CHOR | Obl - Obl | 0.36 | 0.3961 |
| CHLU - CHRE | Obl - Obl | -0.33 | 0.4005 |
| CHOR - CHRE | Obl - Obl | -0.77 | 0.2880 |
| AMSC - CHCI | Obl - Fac | 0.19 | 0.4494 |
| AMSC - CHPE | Obl - Fac | -0.46 | 0.3640 |
| AMSC - CHSP | Obl - Fac | 1.75 | 0.0683 |
| CHLU - CHPE | Obl - Fac | 1.25 | 0.1516 |
| CHLU - CHSP | Obl - Fac | 3.54 | **0.0007** |
| CHOR - CHPE | Obl - Fac | 1.06 | 0.1996 |
| CHOR - CHSP | Obl - Fac | 3.64 | **0.0005** |
| CHRE - CHSP | Obl - Fac | 4.14 | **0.0001** |
| AMSC - WAT | Obl - Env | 3.09 | **0.0032** |
| AMSC - SED | Obl - Env | 2.39 | **0.0213** |
| CHLU - WAT | Obl - Env | 5.11 | **0.0000** |
| CHLU - SED | Obl - Env | 4.38 | **0.0000** |
| CHOR - WAT | Obl - Env | 5.53 | **0.0000** |
| CHOR - SED | Obl - Env | 4.68 | **0.0000** |
| CHRE - WAT | Obl - Env | 5.95 | **0.0000** |
| CHRE - SED | Obl - Env | 5.15 | **0.0000** |
| AMSC - CTFL | Obl - Gra | 2.20 | **0.0306** |
| AMSC - CTST | Obl - Gra | 2.16 | **0.0323** |
| CHLU - CTFL | Obl - Gra | 4.00 | **0.0002** |
| CHLU - CTST | Obl - Gra | 3.83 | **0.0003** |
| CHOR - CTFL | Obl - Gra | 4.16 | **0.0001** |
| CHOR - CTST | Obl - Gra | 3.91 | **0.0002** |
| CHRE - CTFL | Obl - Gra | 4.64 | **0.0000** |
| CHRE - CTST | Obl - Gra | 4.37 | **0.0000** |
| CHCI - CHLU | Fac - Obl | -1.79 | 0.0646 |
| CHCI - CHOR | Fac - Obl | -1.66 | 0.0762 |
| CHCI - CHRE | Fac - Obl | -2.21 | **0.0311** |
| CHPE - CHRE | Fac - Obl | -1.68 | 0.0777 |
| CHCI - CHPE | Fac - Fac | -0.63 | 0.3296 |
| CHCI - CHSP | Fac - Fac | 1.49 | 0.1021 |
| CHPE - CHSP | Fac - Fac | 2.29 | **0.0265** |
| CHCI - WAT | Fac - Env | 2.73 | **0.0088** |
| CHCI - SED | Fac - Env | 2.06 | **0.0388** |
| CHPE - WAT | Fac - Env | 3.74 | **0.0004** |
| CHPE - SED | Fac - Env | 3.00 | **0.0041** |
| CHSP - WAT | Fac - Env | 1.23 | 0.1543 |
| CHSP - SED | Fac - Env | 0.50 | 0.3619 |
| CHCI - CTFL | Fac - Gra | 1.91 | 0.0528 |
| CHCI - CTST | Fac - Gra | 1.90 | 0.0524 |
| CHPE - CTFL | Fac - Gra | 2.75 | **0.0086** |
| CHPE - CTST | Fac - Gra | 2.66 | **0.0101** |
| CHSP - CTFL | Fac - Gra | 0.46 | 0.3691 |
| CHSP - CTST | Fac - Gra | 0.55 | 0.3570 |
| WAT - SED | Env - Env | -0.82 | 0.2772 |
| CTFL - WAT | Gra - Env | 0.72 | 0.3000 |
| CTFL - SED | Gra - Env | -0.01 | 0.4968 |
| CTST - WAT | Gra - Env | 0.53 | 0.3557 |
| CTST - SED | Gra - Env | -0.14 | 0.4625 |
| CTFL - CTST | Gra - Gra | 0.12 | 0.4608 |

**Table S3:** **Mean relative abundances of genes associated with five Symbiodiniaceae genera identified in this study per sample type (e.g., *Amanses scopas* feces) and overall sample category (e.g., obligate corallivores) (Figure 3).** Included samples (and sample sizes) for each sample category are as follows: Corals: *Acropora hyacinthus*, ACR (11); *Pocillopora* spp. = *Pocillopora* species complex, POC (12); *Porites lobata* spp. = *Porites lobata* species complex, POR (12). Obligate corallivores: *Amanses scopas,* AMSC (7)*; Chaetodon lunulatus,* CHLU (8); *Chaetodon ornatissimus,* CHOR (14); *Chaetodon reticulatus,* CHRE (11). Facultative corallivores: *Chaetodon pelewensis,* CHPE (8); *Chaetodon citrinellus,* CHCI (6); and *Chlorurus spilurus* CHSP (8). Grazer/Detritivores: *Ctenochaetus flavicauda,* CTFL (6); and *Ctenochaetus striatus,* CTST (6). Sediment and water: Sediment, SED (12); Water, WAT (7).

| Species | Code | Symbiodiniaceae genus | | | | |
| --- | --- | --- | --- | --- | --- | --- |
|  |  | *Symbiodinium* | *Breviolum* | *Cladocopium* | *Durusdinium* | *Fugacium* |
| **Corals** |  |  |  |  |  |  |
| *Acropora hyacinthus* | ACR | 0.53 | 0.00 | 0.08 | 0.39 | 0.00 |
| *Pocillopora* spp. | POC | 0.00 | 0.00 | 0.89 | 0.11 | 0.00 |
| *Porites lobata* spp. | POR | 0.00 | 0.00 | 0.98 | 0.02 | 0.00 |
|  |  |  |  |  |  |  |
| **Obligate corallivores** |  |  |  |  |  |  |
| *Amanses scopas* | AMSC | 0.01 | 0.00 | 0.72 | 0.27 | 0.00 |
| *Chaetodon lunulatus* | CHLU | 0.00 | 0.00 | 0.87 | 0.13 | 0.00 |
| *Chaetodon ornatissimus* | CHOR | 0.00 | 0.00 | 0.96 | 0.04 | 0.00 |
| *Chaetodon reticulatus* | CHRE | 0.00 | 0.00 | 0.98 | 0.02 | 0.00 |
| Category average |  | 0.00 | 0.00 | 0.91 | 0.09 | 0.00 |
|  |  |  |  |  |  |  |
| **Facultative corallivores** |  |  |  |  |  |  |
| *Chaetodon citrinellus* | CHCI | 0.08 | 0.00 | 0.81 | 0.11 | 0.00 |
| *Chaetodon pelewensis* | CHPE | 0.00 | 0.00 | 0.72 | 0.28 | 0.00 |
| *Chlorurus spilurus* | CHSP | 0.01 | 0.00 | 0.26 | 0.73 | 0.00 |
| Category average |  | 0.03 | 0.00 | 0.58 | 0.39 | 0.00 |
|  |  |  |  |  |  |  |
| **Grazer/detritivores** |  |  |  |  |  |  |
| *Ctenochaetus flavicauda* | CTFL | 0.03 | 0.00 | 0.08 | 0.89 | 0.00 |
| *Ctenochaetus striatus* | CTST | 0.18 | 0.00 | 0.14 | 0.66 | 0.02 |
| Category average |  | 0.10 | 0.00 | 0.11 | 0.78 | 0.01 |
|  |  |  |  |  |  |  |
| **Sediment and seawater** |  |  |  |  |  |  |
| Sediment | SED | 0.15 | 0.02 | 0.66 | 0.13 | 0.04 |
| Water | WAT | 0.19 | 0.02 | 0.70 | 0.08 | 0.00 |
| Category average |  | 0.17 | 0.02 | 0.68 | 0.11 | 0.02 |

**Table S4**: **Results from pairwise PERMANOVA tests on Symbiodiniaceae community composition at the genus level, based on Bray-Curtis distances (Figure 3).** Samples were randomly subsampled from each sample category (n=12 each, see **Supplementary Materials** for sample names). We controlled for false positives with the Benjamini-Hochberg procedure. Significant p-values (<0.05) are bolded. Overall test results: df=6, F=17.3, R^2^=0.58, p=0.001 (PERMANOVA). *Acropora*: *Acropora hyacinthus*; *Pocillopora* spp. *=* *Pocillopora* species complex; *Porites lobata* spp. = *Porites lobata* species complex.

| Comparisons | R^2^ | Adjusted p-value |
| --- | --- | --- |
| Obligate corallivore vs Facultative corallivore | 0.20 | **0.025** |
| Obligate corallivore vs Grazer/detritivore | 0.80 | **0.002** |
| Obligate corallivore vs Sediment and water | 0.14 | **0.020** |
| Obligate corallivore vs *Acropora hyacinthus* | 0.49 | **0.002** |
| Obligate corallivore vs *Pocillopora* spp. | 0.03 | 0.431 |
| Obligate corallivore vs *Porites lobata* spp. | 0.11 | 0.129 |
| Facultative corallivore vs Grazer/detritivore | 0.50 | **0.002** |
| Facultative corallivore vs Sediment and water | 0.09 | 0.117 |
| Facultative corallivore vs *Acropora hyacinthus* | 0.32 | **0.002** |
| Facultative corallivore vs *Pocillopora* spp. | 0.16 | **0.049** |
| Facultative corallivore vs *Porites lobata* spp. | 0.39 | **0.002** |
| Grazer/detritivore vs Sediment and water | 0.51 | **0.002** |
| Grazer/detritivore vs *Acropora hyacinthus* | 0.26 | **0.014** |
| Grazer/detritivore vs *Pocillopora* spp. | 0.82 | **0.002** |
| Grazer/detritivore vs *Porites lobata* spp. | 0.90 | **0.002** |
| Sediment and water vs *Acropora hyacinthus* | 0.28 | **0.002** |
| Sediment and water vs *Pocillopora* spp. | 0.14 | **0.006** |
| Sediment and water vs *Porites lobata* spp. | 0.25 | **0.002** |
| *Acropora* *hyacinthus* vs *Pocillopora* spp. | 0.49 | **0.002** |
| *Acropora* *hyacinthus* vs *Porites lobata* spp. | 0.57 | **0.002** |
| *Pocillopora* spp. vs *Porites lobata* spp. | 0.33 | **0.009** |


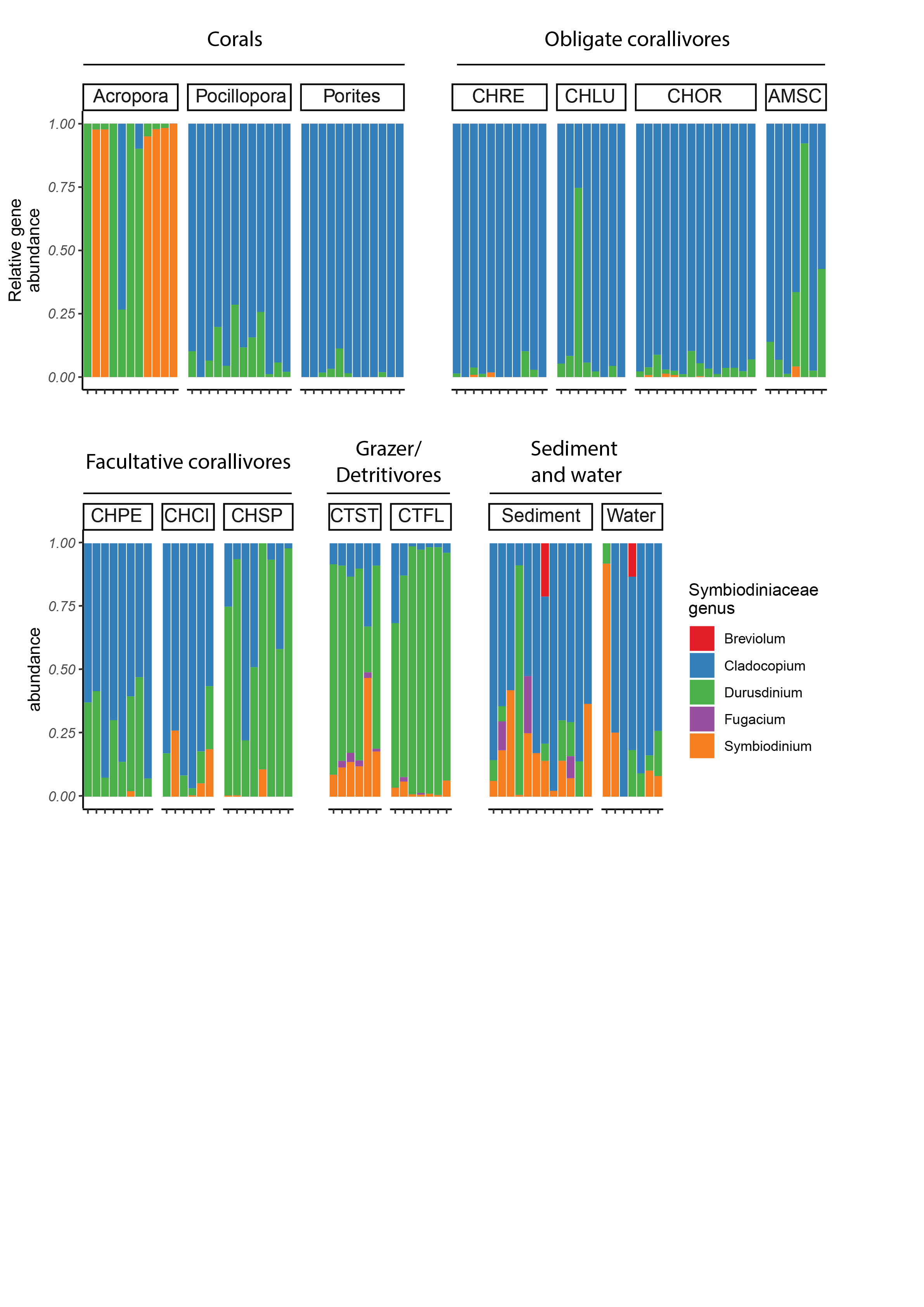
**Figure S2: The communities of Symbiodiniaceae in obligate corallivore (coral-eating animal) feces are most similar to the communities of Symbiodiniaceae in two locally abundant coral species.** Relative abundance of hits to a given Symbiodiniaceae genus in corals, obligate corallivore feces, facultative corallivore feces, grazer/detritivore feces, and reef-associated sediment and water (all included samples had >1,000 reads). Each bar represents one fecal sample from one fish or coral individual, or a separate sediment or water sample. Included samples (and sample sizes) for each sample category in the figure are as follows: Corals: *Acropora hyacinthus*, Acropora (11); *Pocillopora* species complex, Pocillopora (12); *Porites lobata* species complex, Porites (12). Obligate corallivores: *Amanses scopas,* AMSC (7)*; Chaetodon lunulatus,* CHLU (8); *Chaetodon ornatissimus,* CHOR (14); *Chaetodon reticulatus,* CHRE (11). Facultative corallivores: *Chaetodon pelewensis,* CHPE (8); *Chaetodon citrinellus,* CHCI (6); and *Chlorurus spilurus* CHSP (8). Grazer/Detritivores: *Ctenochaetus flavicauda,* CTFL (6); and *Ctenochaetus striatus,* CTST (6). Sediment and water: Sediment, SED (12); Water, WAT (7). For data used see **Additional file 2**.

**Table S5: Overview of values used in bootstrap estimate of reef-scale Symbiodiniaceae dispersal (Figure 4).** Fecal samples were used to calculate mean densities of live Symbiodiniaceae cells and mean fecal densities. Fish densities were calculated from the MCR LTER dataset (<http://mcrlter.msi.ucsb.edu/cgi-bin/showDataset.cgi?docid=knb-lter-mcr.6> accessed February 14, 2020). Data on fecal pellet sizes and egestion rates were collected during *in situ* fish follows. Obl: obligate corallivore; Fac: facultative corallivore. See **Supplementary Methods** for additional details.

| Species | Diet | Live Symbiodiniaceae density (cells g^-1^) | | | Fecal pellet  size (cm) | | | Fecal sample  density (g cm^-1^) | | | Fish density (individuals 250 m^-2^) | | | Egestion rate (h^-1^) | | |  |
| --- | --- | --- | --- | --- | --- | --- | --- | --- | --- | --- | --- | --- | --- | --- | --- | --- | --- |
|  |  |  |  |  |  |  |  |  |  |  |  |  |  |  |  |  |  |
|  |  | N | mean | sd | N | mean | sd | N | mean | sd | N | mean | sd | Estimate |  |  |  |
| CHOR | Obl | 8 | 4.56E+06 | 3.08E+06 | 6 | 5.08 | 2.59 | 3 | 0.14 | 0.02 | 8 | 9.88 | 3.52 | 1.34 |  |  |  |
| CHRE | Obl | 8 | 9.22E+06 | 1.94E+06 | 2 | 4.50 | 0.50 | 3 | 0.18 | 0.03 | 8 | 5.25 | 2.66 | 1.08 |  |  |  |
| CHCI | Fac | 6 | 1.22E+06 | 1.78E+06 | 4 | 2.75 | 1.03 | 4 | 0.04 | 0.06 | 8 | 1.13 | 1.54 | 0.97 |  |  |  |
